# Inhibition of the T cell oxygen sensing machinery promotes anti-tumor efficacy

**DOI:** 10.1186/2051-1426-3-S2-O1

**Published:** 2015-11-04

**Authors:** David Clever, Rahul Roychoudhuri, Nicholas P Restifo

**Affiliations:** 1 Surgery Branch, National Institutes of Health, National Cancer Institute, Bethesda, MD, USA

## 

Mechanisms of immune tolerance can also be co-opted by tumors to subvert anti-tumor effector T cell responses. The lung represents a unique anatomic tissue of pronounced immune tolerance where T lymphocytes do not mount effector responses under steady state conditions despite continual exposure to innocuous foreign antigens. We hypothesized that a cell intrinsic mechanism by which T lymphocyte behavior is modulated to promote local immune suppression within the lungs can be inhibited to promote anti-tumor T effector responses. Here, we demonstrate that the family of prolyl-hydroxylase (Phd) proteins promote T cell tolerance in the lung and limit anti-tumor T cell responses throughout the body. Prolyl-hydroxylase isoforms 1, 2, and 3 functioned redundantly as oxygen sensors in T lymphocytes and enabled environmental oxygen to limit effector differentiation in vitro and in vivo. Mice with a T-cell specific deletion of all three Phd proteins (*Phd-tKO*) developed spontaneous Th1-mediated immunopathology in the lungs. *Phd-tKO* CD4^+^ T lymphocytes activated under normoxic conditions or wild-type T cells activated under oxygen-deprivation conditions exhibited reduced specification into T regulatory cells (Treg) and instead defaulted to a Th1 program as revealed by whole transcriptome RNA-sequence profiling and induction of Tbet and IFNg protein expression. This predisposition to effector cell specification in the absence of Phd protein expression or under oxygen deprivation was dependent on the functional expression of the hypoxia inducible proteins HIF1a and HIF2a. Importantly, genetic or pharmacologic inhibition of the Phd proteins in tumor antigen-specific CD4^+^ T cells promoted anti-tumor efficacy in murine models of adoptive cell transfer immunotherapy as indicated by increased tumor clearance and improved overall survival of tumor-bearing mice. These findings establish the oxygen sensing Phd proteins as a T cell intrinsic regulatory node in immune homeostasis that may be targeted genetically or pharmacologically to promote anti-tumor immune responses.

